
JOURNAL INFORMATION
==============================
NLM Title Abbreviation: ScientificWorldJournal
Journal ID: ScientificWorldJournal
NLM Journal ID: 101131163
Journal ID: TSWJ

The Scientific World Journal

ISSN: 2356-6140
EISSN: 1537-744X
Publisher: Wiley

ARTICLE INFORMATION
==============================
PMCID: PMC5956466
PMID: 15349550
DOI: 10.1100/tsw.2004.75
Article ID: 271020
Article version: 1
Subjects: Research Article

Self-Retraction Clamp for Dissection of the Posterior Prostatic Fossa During Radical Retropubic Prostatectomy

Olumi Aria F.
Brigham & Women's Hospital, Harvard Medical School, Boston, MA 02115, USA
Academic Editor: Anthony Atala

Publication date: 2004
Electronic publication date: 2004 Jun 7
Volume: 4
First page: 260
Last page: 262
Copyright: Copyright © 2004 Aria F. Olumi.
Copyright year: 2004
License: This is an open access article distributed under the Creative Commons Attribution License, which permits unrestricted use, distribution, and reproduction in any medium, provided the original work is properly cited.
License URL: https://creativecommons.org/licenses/by/3.0/

==============================
Articles
*TheScientificWorldJOURNAL* (2004) 4 (S1), 260–262
ISSN 1537-744X; DOI 10.1100/tsw.2004.75

### Self-Retraction Clamp for Dissection of the Posterior Prostatic Fossa During Radical Retropubic Prostatectomy

Aria F. Olumi, M.D.
*Brigham & Womenís Hospital, Harvard Medical School, Boston, MA 02115*

**E-mail**: aolumi@caregroup.harvard.edu

*Previously published in the Digital Urology Journal*

---

**DOMAIN**: urology

---

### INTRODUCTION

Dissection of the posterior prostatic fossa is a critical part of radical retropubic prostatectomy (RRP). Two main goals during this portion of surgery are 1. to identify the correct plane on the anterior wall of the rectum to ensure all layers of Denonvilliers fascia are excised, and 2. to avoid trauma to the neurovascular bundle[1]. After resection of the anterior urethra, the Foley catheter is brought through, clamped and divided, followed by incision of the posterior urethral wall. With the aid of a hemostat clamp on the divided Foley catheter, the prostate is retracted cranially to dissect the lateral pelvic fascia and identify the neurovascular bundle. However, manual cranial retraction of the prostate is imprecise; too much retraction can damage the neurovascular bundle, and too little retraction does not delineate the fascial planes between the prostate and the rectum. Therefore, a self-retraction prostate clamp was designed to enable the surgeon to apply constant traction on the Foley catheter during dissection of the posterior prostatic fossa during RRP. (The prostate self-retraction clamp can be purchased from: J Hugh Knight Instrument Company, 226 S. Villere St., New Orleans, LA 70112, 504-524-2797.)

### TECHNIQUE

The clamp is divided into three portions, segments A and B connected by a 20 cm ball-bearing chain (Figure 1). Prior to resection of the Foley catheter, the catheter is clamped with segment A of the retraction device. Segment A of the retraction clamp secures the catheter without sliding and the toothed segment (arrow Figure 1) limits the maximum applied pressure on the Foley catheter in order to avoid damaging the balloon port. Segment B is attached to the Bookwalter ring or the Balfour retractor. The tension on the chain is adjusted by cocking the ball-bearing chain in the notched screw of segment B (dashed arrow Figure 1, and Figure 2). By using the self-retraction device, the clamp is adjusted on the Bookwalter or the Balfour ring only twice (once for each posterior prostatic pedicle). The rest of the adjustments simply are by varying the length in the "ball-bearing" chain as the dissection of the posterior prostatic fossa is continued cranially. The length of the ball-bearing chain is adjusted to vary the applied

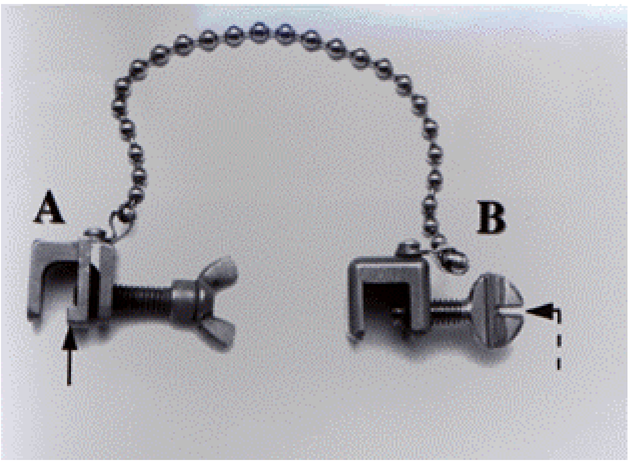

**FIGURE 1**

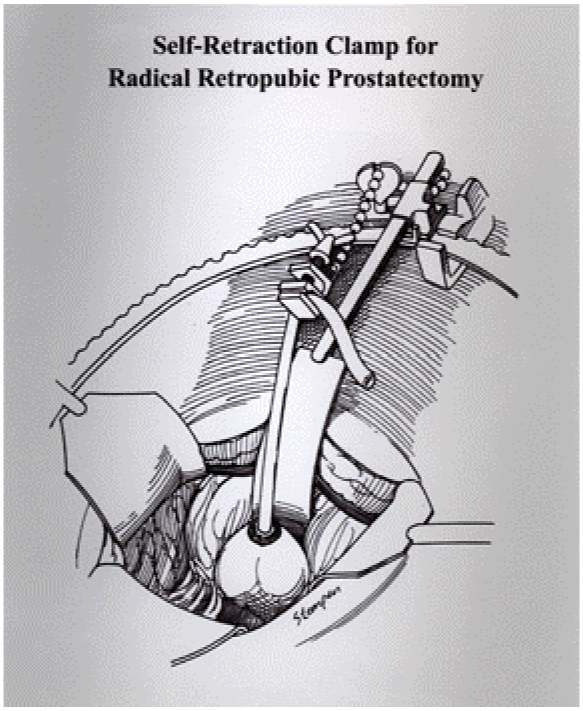

**FIGURE 2**

traction on the lateral pelvic fascia and the neurovascular bundles, as resection of the prostate gland proceeds cranially. In addition to applying constant steady traction on the posterior prostate gland during RRP, this traction device frees the surgeon's hand from manually retracting the Foley catheter.

### CONCLUSION

In conclusion, a self-retraction clamp has been designed to apply constant steady traction on the prostate gland during dissection of the posterior prostatic fossa. The constant adjustable traction delineates the surgical planes between the prostate and the rectum. In addition, the surgeonís hand is freed from manually retracting the Foley catheter during dissection of the posterior prostatic fossa.

### REFERENCE

1. Walsh, P.C.: Anatomic Radical Retropubic Prostatectomy: In: Campbell's Urology 7th edition. Edited by P. C. Walsh, A. B. Retik, T. A. Stamey, and E. D. Vaughan Jr., Philadelphia: W. B. Saunders, vol. 3, chapt. 86, pp. 2565-88, 1997.

**This article should be referenced as follows:**

Olumi, A.F. (2004) Self-retraction clamp for dissection of the posterior prostatic fossa during radical retropubic prostatectomy. *TheScientificWorldJOURNAL* **4 (S1)**, 260–262.

**Handling Editor:**

Anthony Atala, Principle Editor for *Urology* — a domain of *TheScientificWorldJOURNAL*.
